# Supplementary material for: A New Laboratory Workflow Integrating the Free Light Chains Kappa Quotient into Routine CSF Analysis
Source: Biomolecules. 2022 Nov 15;12(11):1690. doi: 10.3390/biom12111690 (PMC9687331; doi:10.3390/biom12111690)
Supplement: Supplementary file 1 [file biomolecules-12-01690-s001.zip › supplemental_material_biomolecules_Figure S1.pdf]

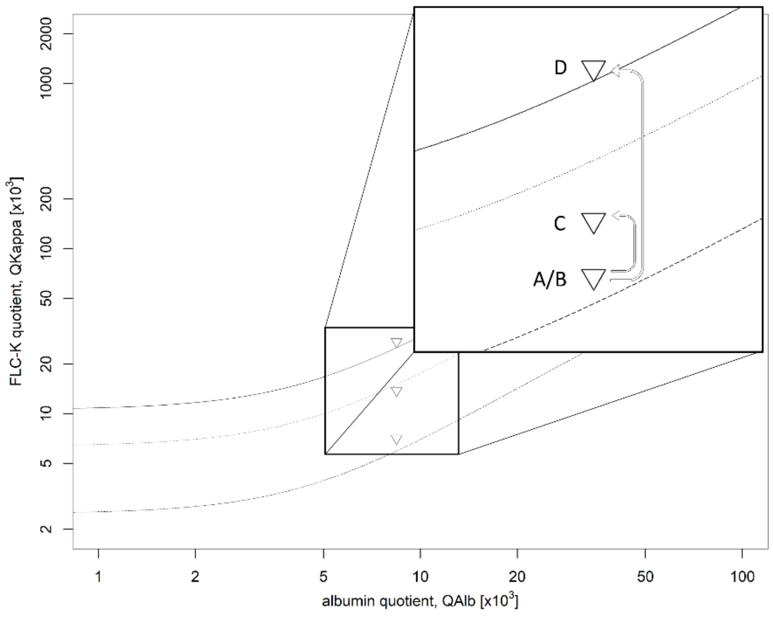

Figure S1. Data of the hypothetic patient in a double logarithmic diagram in case of normal- and increased serum values as well as with- or without intrathecal FLCκ synthesis. Patient A/B: CSF serum quotient of normal and increased serum and CSF values (table 1.1 Patient 1-A, Patient 1-B). Patient C: Increased serum values and intrathecal synthesis of FLCκ. Patient D: normal serum values with intrathecal FLCκ synthesis. FLCκ free light chains kappa, Q quotient, Alb albumin
