# Supplementary material for: A New Laboratory Workflow Integrating the Free Light Chains Kappa Quotient into Routine CSF Analysis
Source: Biomolecules. 2022 Nov 15;12(11):1690. doi: 10.3390/biom12111690 (PMC9687331; doi:10.3390/biom12111690)
Supplement: Supplementary file 1 [file biomolecules-12-01690-s001.zip › supplemental_material_biomolecules_Table S1.pdf]

Table S1. CSF-serum values as well as quotient and resulting IF of the hypothetic patient in case of normal- and increased FLCκ serum values as well as with- or without intrathecal FLCκ synthesis.

	FLCκ CSF (mg/L)	FLCκ serum (mg/L)	QFLCκ	IF FLCκ (%)
Patient 1-A	0.13	18.3	7.10	0
Patient 1-B	0.39	54.9	7.10	0
Patient 1-C	0.76	54.9	13.84	0
Patient 1-D	0.5	18.3	27.32	7.3
